# Associations between ultrasound measurements and hematochemical parameters for the assessment of liver metabolic status in Holstein–Friesian cows

**DOI:** 10.1038/s41598-021-95538-x

**Published:** 2021-08-11

**Authors:** Diana Giannuzzi, Rossella Tessari, Sara Pegolo, Enrico Fiore, Matteo Gianesella, Erminio Trevisi, Paolo Ajmone Marsan, Michele Premi, Fiorenzo Piccioli-Cappelli, Franco Tagliapietra, Luigi Gallo, Stefano Schiavon, Giovanni Bittante, Alessio Cecchinato

**Affiliations:** 1 Department of Agronomy, Food, Natural Resources, Animals and Environment (DAFNAE), University of Padua, Viale dell’Università 16, 35020 Legnaro, Padua, Italy; 2 Department of Animal Medicine, Productions and Health (MAPS), University of Padua, Legnaro, Padua, Italy; 3 Department of Animal Science, Food and Nutrition (DIANA), Università Cattolica del Sacro Cuore, Piacenza, Italy; 4 Nutrigenomics and Proteomics Research Center (PRONUTRIGEN), Università Cattolica del Sacro Cuore, Piacenza, Italy

**Keywords:** Chemical biology, Biomarkers

## Abstract

Metabolic disorders, including hepatic lipidosis and ketosis, severely affect animal health status and welfare with a large economic burden in dairy herds. The gold standard for diagnosing hepatic lipidosis is the liver biopsy, which is impractical and invasive for the screening at farm level. Ultrasound (US) imaging is a promising technique for identifying liver dysfunction, but standardized specifications in physiological conditions are needed. Herein, we described the features of four US measurements, namely the liver predicted triacylglycerol (pTAG) content, liver depth (LD), and portal vein area (PVA) and depth (PVD) and we investigated their associations with a set of hematochemical (HC) indicators in 342 clinically healthy Holstein Friesian dairy cows. Liver pTAG content was negatively associated with hematocrit and positively with globulin, whereas PVA was negatively associated with thiol group levels, and LD positively with ceruloplasmin. We found significant interactions between some HC parameters and parity: in particular, creatinine, thiol groups and globulin for PVA, and aspartate aminotransferase, paraoxonase and ceruloplasmin for PVD. This study offers new insights on variations in liver function occurring after calving and pave the way for the potential use of minimally invasive techniques for prompt detection of metabolic disorders in dairy herds.

## Introduction

Metabolic disorders in early lactation severely affects dairy cow health status and welfare with a large economic burden^1,2^. Parturition and the onset of lactation put an enormous physiological stress on the cow’s homeostatic processes^3,4^. When dietary energy intake is low and/or energy requirements rise up, non-esterified fatty acids (NEFA) are mobilized from the adipose tissue. If blood NEFA levels are elevated for prolonged periods and liver activities are overburdened, the excess NEFA may accumulate as triacylglycerol (TAG) in the hepatic tissue^5,6^, resulting in liver dysfunction and development of ketosis. Hepatic lipidosis, namely the fatty infiltration of hepatic tissue, is the most common metabolic disorder in high-yielding dairy cows^7^ and is closely linked to ketosis^8,9^, and early detection of these metabolic disorders is crucial to improve dairy production and herd profitability.

The gold standard test for diagnosing hepatic lipidosis is histological determination of TAG contents in the liver^10^. However, liver biopsies are impractical to perform on farm due to the time required for acquisition and analysis^11^, the risk of infection and hemorrhage^12^, and the discomfort the procedure causes the cows^13^. Transcutaneous ultrasound (US) imaging of the liver has been proposed as a noninvasive method, but metabolic alterations, including lipidosis, are difficult to detect as they give rise to changes in hepatic texture^14^ that may be imperceptible at US evaluation. Furthermore, while US images are sensitive to tissue structure, they are also sensitive to the features and settings of the US scanner. To overcome these limitations, quantitative methods that extract the relevant information from the US images have been developed, including texture analysis^15,16^. Diverse studies have focused on the use of texture analysis of liver US images to predict TAG (pTAG) content as an indicator of hepatic lipidosis in dairy cows^11,17–19^. In addition, preliminary studies have explored the benefits of using US measurements of liver dimensions and liver-related anatomical structures (*i.e.* liver depth, portal vein diameter and depth) to identify hepatic alterations^20–22^.

Alongside US imaging, the evaluation of blood biochemical indicators is a widely-established, minimally-invasive analytical method for identifying animals with clinical disease^23^. In the last years, specific blood reference panels of hematochemical (HC) indicators have been developed to identify early hepatic metabolic dysfunctions in clinically healthy animals^24–26^. Indeed, profiling blood biomarkers of lipomobilization, liver functionality, oxidative stress and inflammation may help in monitoring and preventing subclinical ketosis and early fatty liver conditions^9^.

Although promising results emerged from the use of US imaging as a diagnostic tool for hepatic lipidosis in overt clinical conditions, there is a lack of information on liver US measurements and the patterns of liver US-predicted parameters in clinically healthy dairy cows. Moreover, their variations in relationship with HC indicators in physiological conditions has never been investigated which is the first step towards their possible implementation as screening tools.

Within this context, in the present study we first describe the physiological features and study the variability of US liver pTAG, liver depth (LD), and portal vein area (PVA) and depth (PVD) traits. Second, we investigate the associations between US traits and a set of HC parameters, including indicators of energy metabolism, liver oxidative stress/damage and innate immune response, and minerals. The study was conducted on a population of 342 clinically healthy Holstein Friesian dairy cows in early lactation as a basis for further characterization of alterations in liver metabolic function.

## Results

### Descriptive statistics

Descriptive statistics for the US and HC parameters are reported in Supplementary Table S1.

At pTAG content evaluation, the 1% of cows (n = 4) had critical values (> 100 mg/g), as defined by^18^. No other concomitant alterations in blood parameters were observed in these cows, and the remaining US measurements were in the physiological range. In total, 2% of cows (n = 7) had BHBA concentrations greater than 1.2 mmol/L and NEFA concentrations greater than 0.70 mmol/L (n = 6) and were distributed in all the DIM classes. No concomitant alterations in US measurements were observed in these cows. No animals had serum urea concentration below the optimal range (< 1.7 mmol/L) or hypocalcemia (< 2.0 mmol/L).

All the US parameters showed a normal distribution, with very low skewness values (Supplementary Figure S1). The pTAG averaged 69.52 mg/g, with a coefficient of variation close to 15%. PVA averaged 1115.65 mm^2^, PVD 130.77 mm, and LD 149.12 mm. The ranges of all the parameters were consistent with physiological conditions^18,20^.

### Sources of variation in US traits

The results from the linear mixed models used to characterize the variability in US traits are reported in Table 1.

**Table 1:** Results from linear mixed model (*P* values) and least square means for predicted liver triacylglycerol (pTAG), portal vein area (PVA), portal vein depth (PVD) and liver depth (LD).

Traits	DIM^a^						Parity			Herd/date^b^	RMSE^c^
	*P* value	Least square means					*P* value	Least square means			
		1	2	3	4	5		Primiparous	Multiparous		
pTAG, mg/g	**0.0007**	65.0	68.7	71.4	73.1	70.3	**0.0049**	66.4	73.0	8.72	9.75
PVA, mm^2^	0.8401	1110	1111	1149	1107	1149	0.1295	1099	1151	0.35	279.20
PVD, mm	0.4210	131	130	133	132	135	**0.0070**	128	136	7.41	12.17
LD, mm	0.7443	148	149	151	151	149	**0.0038**	145	154	7.45	12.09

*P* values < 0.1 are highlighted using bold.

^a^DIM classes are: class 1 ≤ 30; 30 < class 2 ≤ 60; 60 < class 3 ≤ 90; 90 < class 4 ≤ 120; class 5 > 120.

^b^Variability explained by the random factor herd/date is expressed as a percentage.

^c^*RMSE* root mean square error.

This is the first time, to our knowledge, that the effects of DIM and parity as sources of variation in US measurements, and the effects of herd combined with the date of liver measurement have been evaluated. In our mixed models, the herd/date random effect explained nearly 10% of the total variation for all the US parameters across the different models.

**Figure 1:**
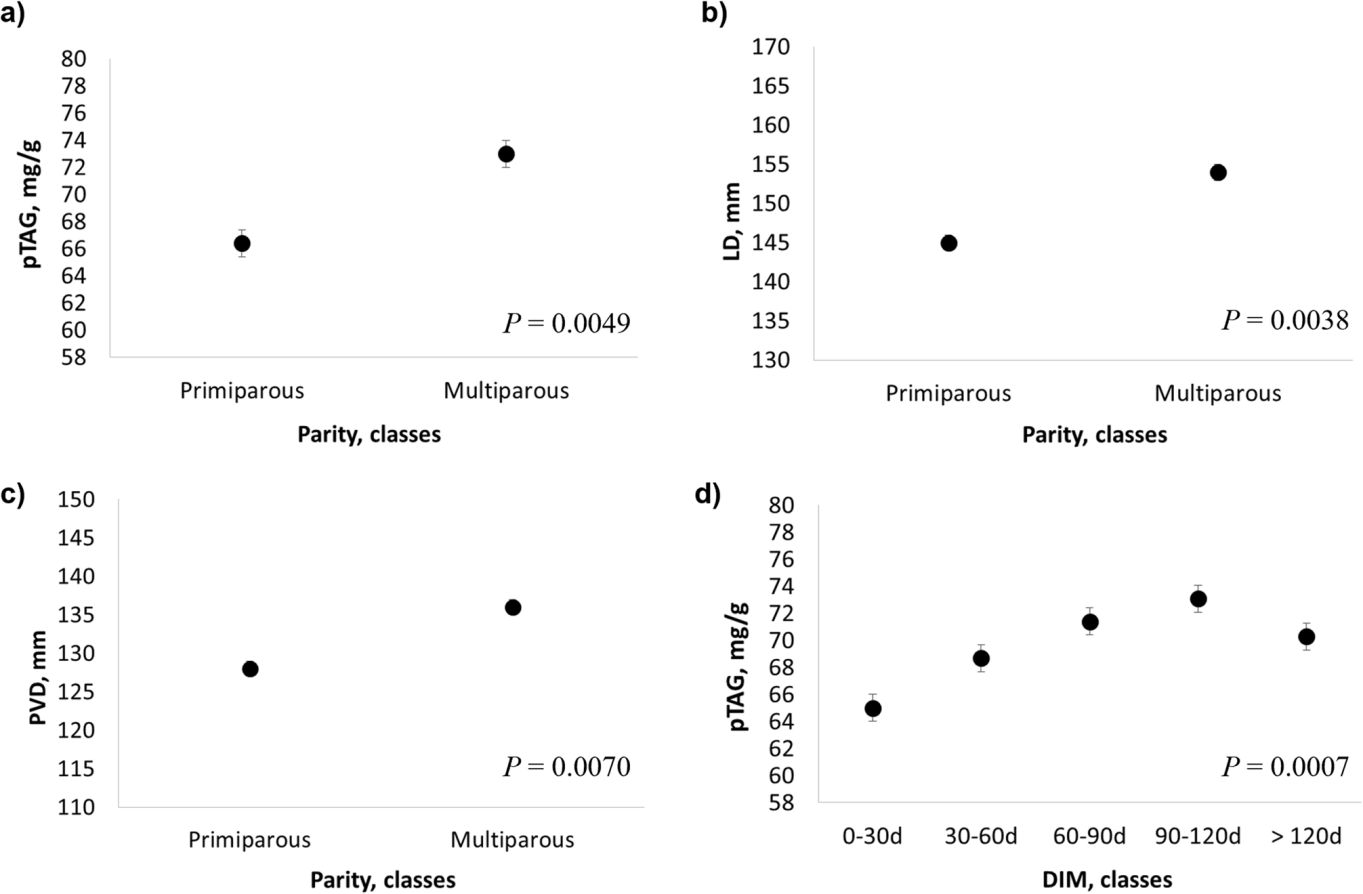
Least squares means (LSM) for (**a**, **d**) predicted liver triacylglycerol (pTAG), (**b**) portal vein depth (PVD) and (**c**) liver depth (LD) across parity and days in milk (DIM) classes. Black dots indicate the LSM and error bars indicate the standard error.

Parity significantly affected pTAG (*P* = 0.0049), PVD (*P* = 0.0070) and LD (*P* = 0.0038), with a general increase from first parity onwards (Fig. 1a–c). The pTAG was also affected by DIM (*P* = 0.0007), with a progressive increase until 120 days of lactation and a slight decrease thereafter (Fig. 1d).

### Associations between US parameters and hematocrit and energy-related blood metabolites

**Figure 2:**
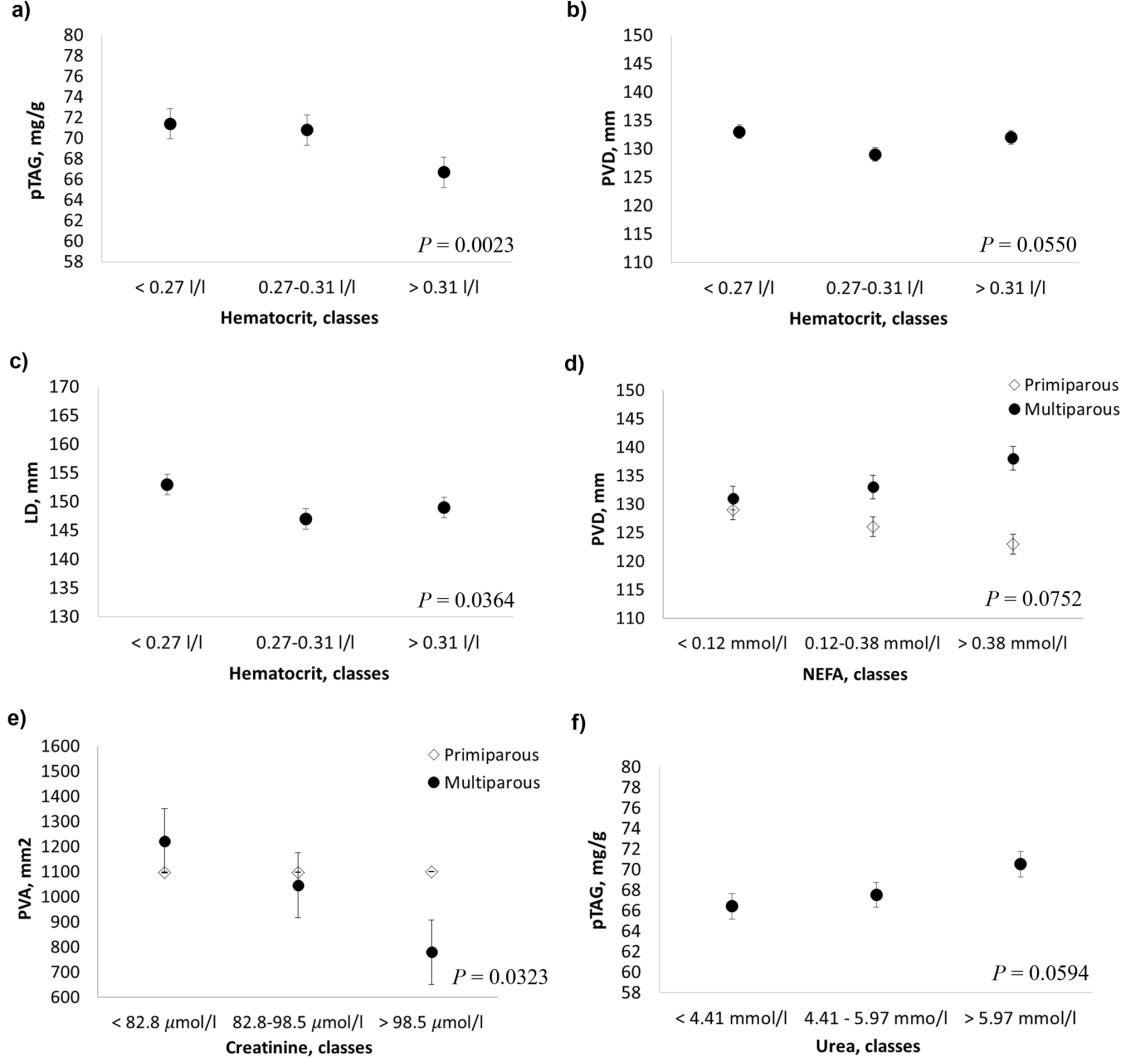
Least squares means (LSM) for predicted liver triacylglycerol (pTAG), portal vein area (PVA), portal vein depth (PVD) and liver depth (LD) across the classes of hematocrit, energy-related hematic metabolites and hematic minerals. (**a**–**c**) hematocrit; (**d**) non esterified fatty acid (NEFA); (**e**) creatinine; (**f**) urea. Black dots/rhombus indicate the LSM and error bars indicate the standard error.

The results from the linear mixed models used to assess the association between US and hematocrit and energy-related metabolites are reported in Table 2 and Fig. 2. There was a significant decrease in the pTAG content and LD with increasing values of hematocrit (*P* = 0.0023 and *P* = 0.0364, respectively; Fig. 2a, b), and the same trend was also observed for PVD (*P* = 0.0550; Fig. 2c).

**Table 2:** Results from linear mixed model (*P* values) for predicted liver triacylglycerol (pTAG), portal vein area (PVA), portal vein depth (PVD) and liver depth (LD).

Traits^a^	df^b^	pTAG, mg/g		PVA, mm^2^		PVD, mm		LD, mm	
		*P* value	RMSE^c^	*P* value	RMSE^c^	*P* value	RMSE^c^	*P* value	RMSE^c^
**Hematochemical parameters**									
Hematocrit, l/l	2	**0.0023**	9.49	0.3182	277.89	**0.0550**	11.98	**0.0364**	11.80
**Energy-related metabolites**									
Glucose, mmol/l	2	0.7754	9.67	0.5966		0.4352	12.10	0.3835	11.94
Glucose × DIM^d^		–	–	**0.0196**	268.03	–	–	–	–
Cholesterol, mmol/l	3	0.6190	9.65	0.4730	277.60	0.5515	12.07	0.4398	11.93
NEFA, mmol/l	2	0.5428	9.69	0.2276	276.66	**0.0855**		0.7343	11.96
NEFA × Parity^e^		–	–	–	–	**0.0752**	11.95	–	–
BHBA, mmol/l	2	0.5874	9.67	0.1912	277.21	0.1666	12.03	0.4383	11.94
Urea, mmol/l	2	**0.0594**	9.55	0.8598	278.69	0.2456	12.02	0.3564	11.91
Creatinine µmol/l	2	0.2588	9.66	0.1936		0.3915	12.05	0.1235	11.86
Creatinine × Parity^e^		–	–	**0.0323**	273.56	–	–	–	–
**Liver function/hepatic damage**									
AST/GOT, U/l	1	0.6647	9.68	**0.0749**	277.28	**0.0301**		0.4778	11.96
AST/GOT × DIM^d^		**0.0310**	9.51	0.0477	272.16	**0.0074**	11.70	0.0427	11.72
AST/GOT × Parity^e^		–	–	–	–	**0.0151**		–	–
GGT, U/l	2	**0.0777**	9.59	0.1217		0.3747	12.06	**0.0972**	11.86
GGT × Parity^e^		–	–	**0.0663**	276.02	–	–	–	–
BILt, μmol/l	1	0.6292	9.68	0.9062	278.84	0.2319		**0.0257**	11.84
BILt xDIM^d^		–	–	**0.0640**	274.38	**0.0769**	11.76	–	–
BILt × Parity^e^		–	–	–	–	**0.0971**		–	–
Albumin, g/l	2	**0.0927**	9.61	0.2597	277.51	0.6019	12.08	0.7118	11.95
ALP, U/l	2	0.9615	9.68	0.7195	278.52	0.6231	12.08	0.9804	11.97
PON, U/ml	2	0.5463	9.66	0.4928	278.14	**0.0228**		0.1088	
PON × Parity^e^		–	–	–	–	**0.0138**	11.85	**0.0958**	11.80

*P* values < 0.1 are highlighted using bold.

^a^NEFA, non-esterified fatty acids; BHBA, β-Hydroxybutyric acid; AST, aspartate aminotransferase; GGT, γ-glutamyl transferase; BILt, total bilirubin; ALP, alkaline phosphatase; PON, paraoxonase. Linear models for pTAG, PVA, PVD and LD always considered the individual sources of variations, namely days in milk (DIM), parity and herd/date and, one at the time, all the hematic variables (in classes). Where *P* values were < 0.1, interactions between DIM and/or parity and hematic variables are reported.

^b^df = the degrees of freedom depend on the number of classes in which hematochemical parameters have been discretized.

^c^*RMSE* root mean square error.

^d^DIM is constituted by 5 classes: class 1 ≤ 30; 30 < class 2 ≤ 60; 60 < class 3 ≤ 90; 90 < class 4 ≤ 120; class 5 > 120.

^e^Parity is constituted by two classes: primiparous and multiparous.

Total cholesterol and β-hydroxybutyric acid (BHBA) were not associated with the US parameters. Decreasing of glucose in individuals during the peak of lactation (DIM class 3, ranging from 60 to 90 DIM) was associated with increasing of PVA (*P* = 0.0196; Table 2). As showed in Table 2 and depicted in Fig. 2, the interaction with parity was relevant for NEFA (affecting PVD, *P* = 0.0752) and creatinine (affecting PVA, *P* = 0.0323). Indeed, at increasing concentrations of NEFA, PVD increased in multiparous cows and decreased in primiparous cows (Fig. 2d). Conversely, the increase in creatinine was associated with decreasing PVA values in multiparous cows but remained unchanged in primiparous cows (Fig. 2e). The pTAG content, however, was affected by urea concentrations regardless of parity, and an increase in pTAG content in the liver was associated with increased uremia (*P* = 0.0594, Fig. 2f).

### Associations between US parameters and liver function/hepatic damage indicators

**Figure 3:**
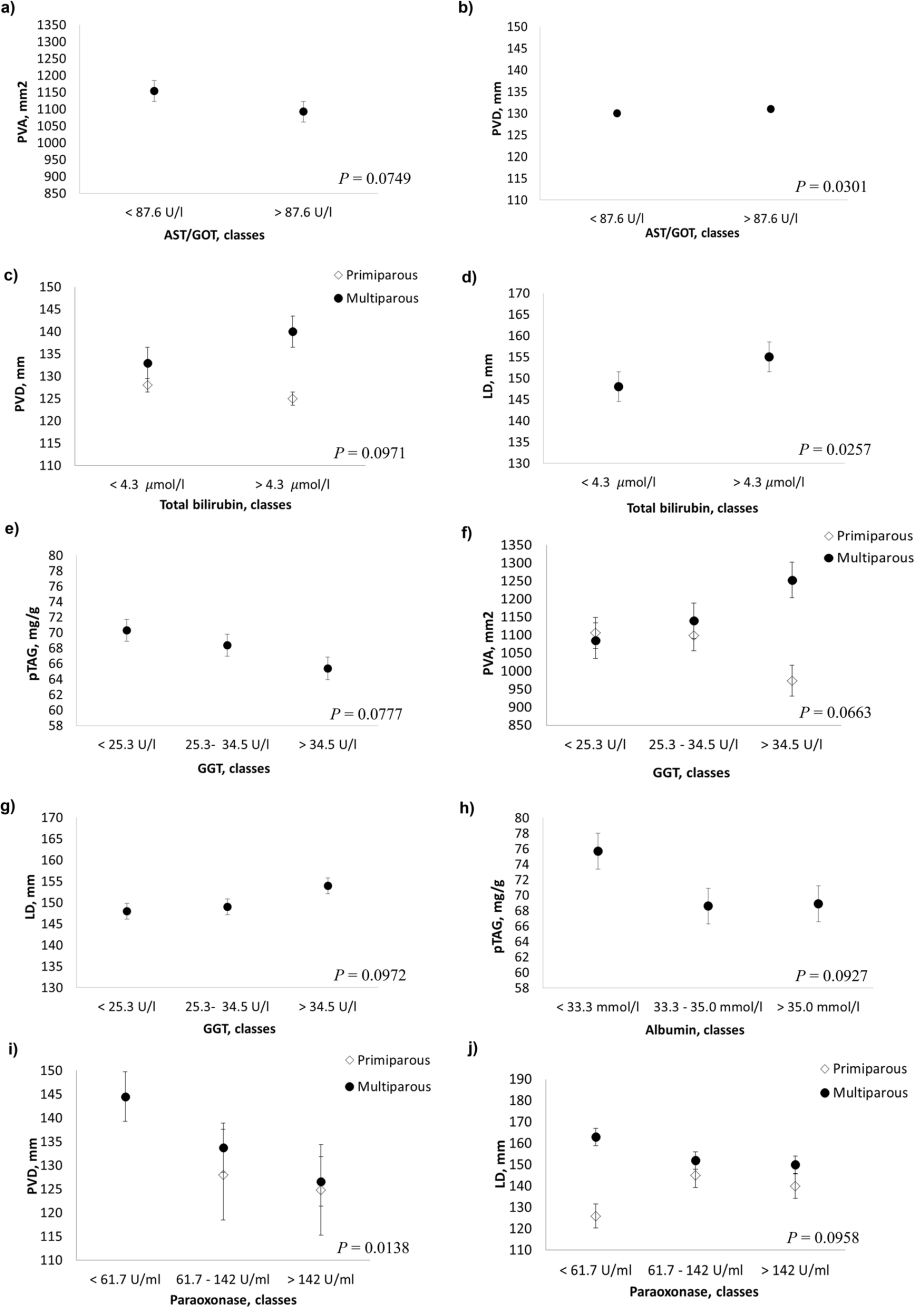
Least squares means (LSM) for predicted liver triacylglycerol (pTAG), portal vein area (PVA), portal vein depth (PVD) and liver depth (LD) across the classes of liver functionality hematic parameters. (**a**, **b**) AST/GOT; (**c**–**e**) GGT; (**f**, **g**) total bilirubin; (**h**) albumin; (**i**, **j**) paraoxonase. Black dots/rhombus indicate the LSM and error bars indicate the standard error.

The results from the linear mixed models used to assess the association between US and liver function/hepatic damage indicators are reported in Table 2 and Fig. 3. Decreasing concentrations of aspartate amino transferase-glutamate oxaloacetate transaminase (AST/GOT) were associated with increasing values of PVA (*P* = 0.0749, Fig. 3a). All the US measurements are affected by the interaction between AST/GOT and DIM; notably, cows in the lactation peak showed an increase of pTAG with increased levels of AST/GOT (*P* = 0.0310 for pTAG, *P* = 0.0477 for PVA, *P* = 0.0074 for PVD, and *P* = 0.0427 for LD).

Moreover, significant interactions with parity were found for AST/GOT and total bilirubin (BILt) affecting PVD (*P* = 0.0151 and *P* = 0.0971, respectively), and for γ-glutamyl transferase (GGT) affecting PVA (*P* = 0.0663); as shown in Fig. 3b, c, increasing concentrations of AST/GOT and BILt were associated with increasing PVD values in multiparous cows and decreasing PVD values in primiparous cows. Increasing concentrations of BILt were associated to a rising trend of PVA and PVD in cows during the peak of lactation (*P* = 0.0640 and *P* = 0.0769, respectively).

Increasing concentrations of GGT were associated with increasing PVA values in multiparous cows and decreasing values in primiparous cows (Fig. 3f). GGT was also related to pTAG content and LD (*P* = 0.0777 and *P* = 0.0972, respectively; Fig. 3e, g): a decrease in GGT concentration was associated with increased pTAG values, while an increase in GGT concentration was associated with increased LD values. LD also significantly increased with higher BILt values (*P* = 0.0257; Fig. 3d). The pTAG content, however, increased with decreasing concentrations of albumin (*P* = 0.0927; Fig. 3h).

The paraoxonase (PON) × parity interaction revealed that at increasing PVD and LD values (*P* = 0.0138 and *P* = 0.0958, respectively), PON increased in multiparous cows and decreased in primiparous cows (Fig. 3i, j).

### Associations between US parameters and liver oxidative stress metabolites

**Figure 4:**
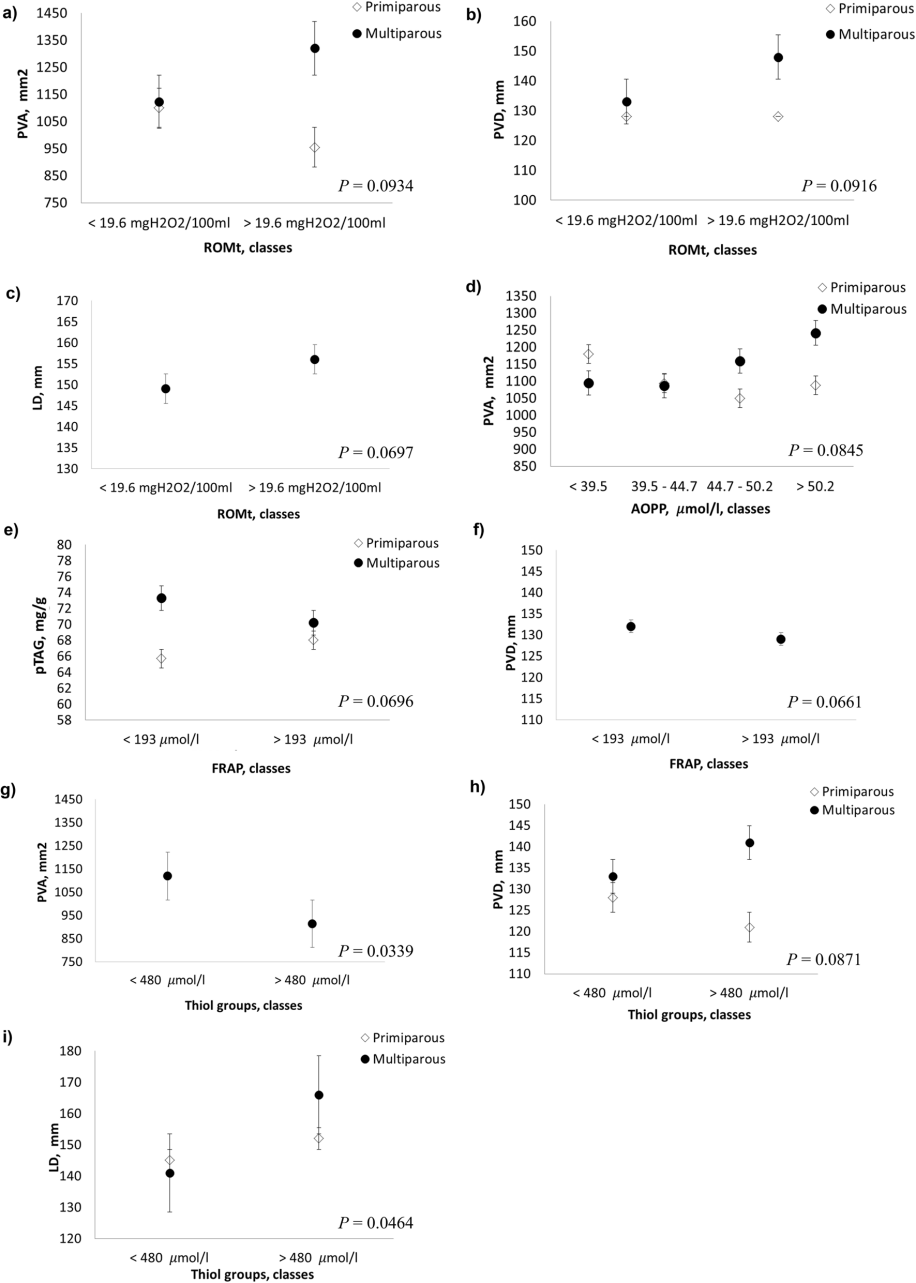
Least squares means (LSM) for predicted liver triacylglycerol (pTAG), portal vein area (PVA) portal vein depth (PVD) and liver depth (LD) across the classes of liver oxidative stress hematic parameters. (**a**–**c**) Total reactive oxygen metabolites (ROMt); (**d**) advanced oxidation protein products (AOPP); (**e**, **f**) Ferric reducing ability of plasma (FRAP); (**g**–**i**) thiol groups. Black dots/rhombus indicate the LSM and error bars indicate the standard error.

Liver oxidative stress metabolites have an impact on several US parameters (Table 3 and Fig. 4). Reactive oxygen metabolites (ROMt) interacted with parity in relationship to portal vein parameters: at increasing values of PVA (*P* = 0.0934) and PVD (*P* = 0.0916), the concentrations of ROMt increased in multiparous cows and decreased in primiparous cows (Fig. 4a, b). Furthermore, the increase in ROMt increased LD (*P* = 0.0697; Fig. 4c). Increasing concentrations of advanced oxidation protein products (AOPP), especially the 2nd, 3rd, and 4th classes, were associated with an increase in PVA in multiparous cows, whereas the opposite trend was observed in primiparous cows with PVA values decreasing as AOPP concentrations increased (*P* = 0.0845; Fig. 4d).

**Table 3:** Results from linear mixed model (*P* values) for predicted liver triacylglycerol (pTAG), portal vein area (PVA), portal vein depth (PVD) and liver depth (LD).

Traits^a^	df^b^	pTAG, mg/g		PVA, mm^2^		PVD, mm		LD, mm	
		*P* value	RMSE^c^	*P* value	RMSE^c^	*P* value	RMSE^c^	*P* value	RMSE^c^
**Hematochemical parameters**									
** Oxidative stress metabolites**									
ROMt, mgH_2_O_2_/100 ml	1	0.8339	9.69	**0.0910**		0.2379	12.00	**0.0697**	11.89
ROMt × Parity^e^	3	–	–	**0.0934**	277.40	**0.0916**	12.07	–	–
AOPP, μmol/l	3	0.6001	9.66	**0.0790**		0.6306	12.04	0.4760	11.95
AOPP × Parity^e^		–	–	**0.0845**	274.80	–	–	–	–
FRAP, μmol/l	1	**0.0741**		0.7315	278.79	**0.0661**	11.99	0.3021	11.96
FRAP × Parity^e^	2	**0.0696**	9.71	–	–	–	–	–	–
Thiol groups, μmol/l	1	0.2547	9.67	**0.0339**	276.63	0.1170		0.1242	
Thiol groups × Parity^e^		–	–	–	–	**0.0871**	12.05	**0.0464**	11.87
**Inflammation/innate immunity**									
CP, µmol/l	2	0.7737	9.67	0.8509	278.68	**0.0509**		**0.0078**	11.74
CP × Parity^e^		–	–	–	–	**0.0206**	11.80	–	–
Total protein, g/l	2	**0.0736**	9.59	0.1550		0.2592	12.04	0.5825	11.94
Total protein × Parity^e^		–	–	**0.0709**	275.90	–	–	–	–
Globulin, g/L	2	**0.0077**	9.52	0.1209		0.1849	12.03	0.5489	
Globulin × DIM^d^		–	–	**0.0498**	267.34	–	–	–	–
Globulin × Parity^e^		–	–	**0.0396**		–	–	–	–
Haptoglobin, g/l	1	0.3543	9.67	0.5557	278.67	0.4368	12.10	0.8178	11.97
Mieloperoxidase, U/l	2	0.1257	9.60	0.6970	278.49	0.8113	12.10	0.8555	11.97
*Minerals*									
Calcium, mmol/l	2	0.3546	9.66	0.3390	277.78	0.9693		0.8052	
Calcium × DIM^d^		–	–	–	–	**0.0001**	11.43	**0.0228**	11.52
Phosphorus, mmol/l	2	0.4997	9.66	0.10	278.74	0.6560	12.08	0.3388	11.93
Magnesium, mmol/l	2	0.2993	9.64	0.66	278.19	0.6983	12.09	**0.0797**	
Magnesium × Parity^e^		–	–	–	–	–	–	**0.0634**	11.84
Sodium, mmol/l	2	0.2845	9.64	0.9655	277.89	0.4719	12.03	0.1203	
Sodium × DIM^d^		–	–	–	–	–	–	**0.0547**	11.49
Potassium, mmol/l	2	0.8465	9.68	1.6099	277.26	0.5624	12.05	0.2071	11.89
Chlorine, mmol/l	2	0.2207	9.64	0.4792	278.37	0.6340	12.06	0.3405	11.90
Zinc, μmol/l	2	**0.0710**		0.5755	278.30	0.5521	12.06	0.4974	11.94
Zinc × Parity^e^		**0.0945**	9.52	–	–	–	–	–	–

*P* values < 0.1 are highlighted using bold.

^a^ROMt, total reactive oxygen metabolites; AOPP, advanced oxidation product of protein; FRAP, ferric reducing antioxidant power; CP, ceruloplasmin. Linear models for pTAG, PVA, PVD and LD always considered the individual sources of variations, namely days in milk (DIM), parity and herd/date and, one at the time, all the hematic variables (in classes). Where *P* values were < 0.1, interactions between DIM and/or parity and hematic variables are reported.

^b^df = the degrees of freedom depend on the number of classes in which hematochemical parameters have been discretized.

^c^*RMSE* root mean square error.

^d^DIM is constituted by 5 classes: class 1 ≤ 30; 30 < class 2 ≤ 60; 60 < class 3 ≤ 90; 90 < class 4 ≤ 120; class 5 > 120.

^e^Parity is constituted by two classes: primiparous and multiparous.

At low concentrations of ferric-reducing antioxidant power (FRAP), PVD values were higher (*P* = 0.0661; Fig. 4f). Regarding the pTAG indicator, the interaction with parity revealed opposite trends at decreasing concentrations of FRAP, with pTAG content increasing in multiparous cows and decreasing in primiparous cows (*P* = 0.0696; Fig. 4e). Finally, high thiol groups concentration showed a decrease in PVA (*P* = 0.0339; Fig. 4g), whereas for PVD and LD, the interaction with parity revealed a different trend: with increasing thiol groups concentration PVD/LD measurements increased in multiparous cows, while in primiparous cows PVD decreased and while the LD measurement remained essentially the same (*P* = 0.0871 and *P* = 0.0464, respectively; Fig. 4h, i).

### Associations between US parameters and inflammatory and innate immunity parameters

**Figure 5:**
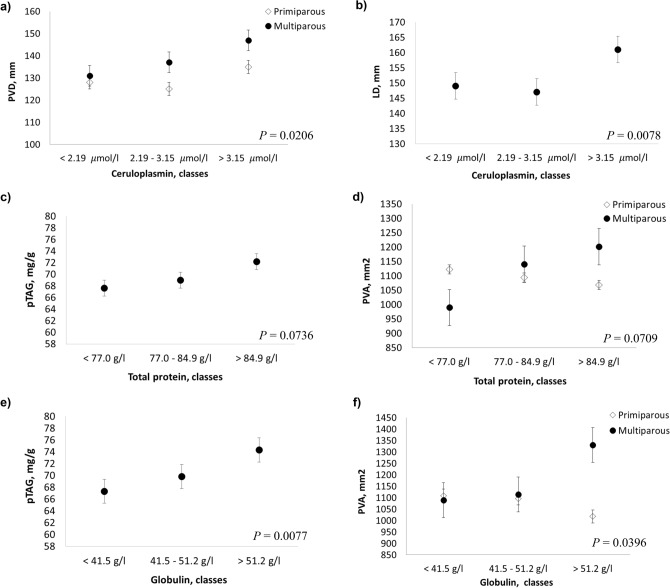
Least squares means (LSM) for predicted liver triacylglycerol (pTAG), portal vein area (PVA) portal vein depth (PVD) and liver depth (LD) across the classes of inflammatory/innate immunity hematic metabolites. (**a**, **b**) Ceruloplasmin; (**c**, **d**) total protein; (**e**, **f**) globulin. Black dots/rhombus indicate the LSM and error bars indicate the standard error.

Haptoglobin and myeloperoxidase had no influence on the US parameters (Table 3). Increasing concentrations of ceruloplasmin (CP) were associated with increasing values of PVD and LD (*P* = 0.02064 and *P* = 0.0078, respectively; Table 3 and Fig. 5a, b). At increasing levels of total protein and globulin, pTAG content increased (*P* = 0.0736 and *P* = 0.0077, respectively; Table 3 and Fig. 5c, e), whereas PVA exhibited opposite trends in multiparous and primiparous cows—increasing in the former and decreasing in the latter (*P* = 0.0709 and *P* = 0.0396, respectively; Table 3 and Fig. 5d, f). The association between PVA and globulin was also significantly affected by the interaction with DIM (*P* = 0.0498; Table 3).

### Associations between US parameters and minerals

Phosphorus, potassium and chlorine showed no association with the US parameters (Table 3). Cows with lower values of calcium during the peak of lactation revealed higher values of PVD and LD (*P* = 0.0001 and *P* = 0.0228, respectively; Table 3). Cows belonging to DIM class 3 and 5 showed a decrease of sodium associated to an increase of LD, whereas cows in the first 30 days of lactation revealed the opposite trend (*P* = 0.0547; Table 3).

**Figure 6:**
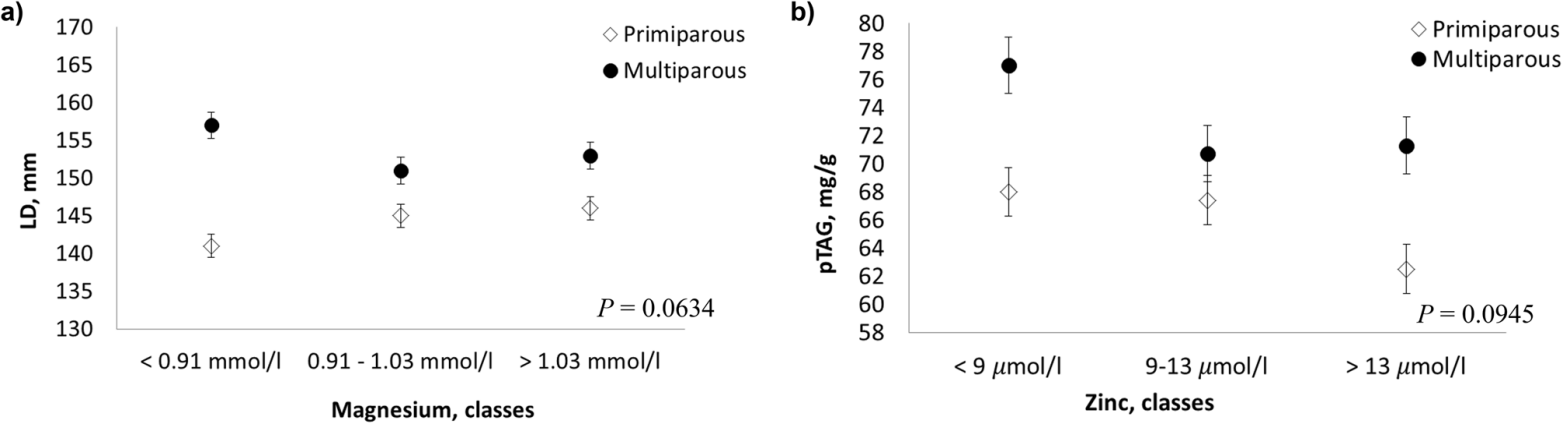
Least squares means (LSM) for predicted liver triacylglycerol (pTAG), and liver depth (LD) across the classes of hematic minerals. (**a**) magnesium; (**b**) zinc. Black dots/rhombus indicate the LSM and error bars indicate the standard error.

Zinc and magnesium exhibited some trends in relation to parity. Increasing levels of magnesium, especially up to a concentration of 1.03 mmol/l, were associated with an increase in LD values in primiparous cows and a decrease in multiparous cows (*P* = 0.0634; Table 3 and Fig. 6a). Increasing levels of zinc, however, were associated with a decrease in pTAG content, which is more pronounced in primiparous cows (*P* = 0.0945; Table 3 and Fig. 6b).

## Discussion

Physiological conditions and/or parameters can be used to assess animal welfare. However, to be able to use these traits as disease indicators, it is essential to define the physiological ranges within which fluctuations do not indicate changes in welfare status (baseline data). Here, we have provided a fingerprint of US measurement indicators of liver metabolic changes in dairy cattle under physiological conditions.

Regarding the different sources of variation in US traits, we found that parity influenced the pTAG content and liver dimensions (expressed as PVD and LD measurements), with multiparous cows having constitutively a larger liver and a higher pTAG content then primiparous cows. Differences in endocrine background, body condition and feed intake capacity between the two parity classes are widely known, and also include liver functionality and lipid mobilization efficiency^27,28^. In our dataset, multiparous cows had lower glucose concentrations than primiparous cows (4.1 *vs* 4.4 mmol/l) and higher NEFA concentrations (0.24 *vs* 0.13 mmol/l). Even though mature animals generally have a better feed intake capacity and body condition, their higher milk production potential makes them less able to adapt to distress due to high metabolic requirements, and they tend to suffer greater negative energy balance and subsequent lipolisis than primiparous cows in early lactation^29^. It is notable that pTAG gradually increased with advancing DIM, reaching the highest values in the 90 to 120 DIM class, then tended to decrease. Body condition scores of dairy cattle are at their minimum 90 to 120 days from calving^30^, but thereafter they start to recover their body reserves. Liver pTAG content may reflect metabolic variations in energy demands during lactation, but further evidence is needed to support this hypothesis.

Finally, the effect of diverse herds and sampling dates explained around 10% of the variability in the population, suggesting that farm management/feeding strategies and sampling dates did not largely affect the estimates.

Notably, cows belonging to the third class of DIM, which correspond to the lactation peak, exhibited more extreme patterns in the relationships between US and HC indicators compared to the other DIM classes, with decreasing levels of glucose, calcium and sodium and increasing AST/GOT and BILt concentrations in association with higher US measurements. These findings highlight the criticality of the lactation peak phase that challenges the organism homeostasis requiring a tremendous adaptability, and where subclinical alterations are the signs of individual inability to balance inputs^31^.

Individuals with lower hematocrit exhibited higher pTAG contents and liver dimensions. In physiological conditions, hematocrit in dairy cows is known to change during lactation and is influenced by sire, season, age^32^, and health status^33,34^. In cows affected by hepatic lipidosis, anemia is an early warning sign^6,35^.

Among the energy-related metabolites, increased levels of NEFA were associated with increased PVD, especially in multiparous cows. NEFA are transported through the portal vein into the liver, where they are metabolized and then enter the bloodstream via the hepatic vein. The increase in the size of the portal vein may arise to cope with the increase in NEFA in early lactation when animals are trying to balance energy intake with energy demands. Indeed, elevated concentrations of NEFA enhance lipogenesis in hepatocytes^36^, which provides the substrate precursors for milk production.

Creatinine was one of the few HC parameters associated with PVA, and was affected by parity, as already shown by^37^. We observed a large reduction in PVA in multiparous cows (from 1221 mm^2^ for creatinine < 82.8 μmol/l, to 779 mm^2^ for creatinine > 98.5 μmol/l), but no relevant trend across creatinine classes in primiparous cows. A possible hypothesis for this inverse relationship between creatinine and PVA might be that the periparturient mobilization of skeletal muscle could be greater in multiparous cows than in primiparous cows due to their higher productivity.

We found an increase in AST associated with an increase in PVD in multiparous cows, suggesting some increased enzymatic activity and blood flow in cows with a greater deployment of muscle reserves for sustaining energy requirements. An increase in blood AST concentrations may be a highly sensitive indicator of the presence of liver damage, even at the subclinical stage^38,39^.

Regarding the other marker of hepatic functionality, GGT, we found contrasting trends for LD (increasing) and pTAG content (decreasing). While the increase in GGT concentrations in association with a larger liver (increased LD) might suggest a predisposition to liver damage and metabolic dysfunction, as described by previous studies^6,40,41^, the decrease in GGT concentrations in association with high pTAG contents probably reflects the animals’ physiological responses to the early phase of lactation, where GGT values decrease to reach minimum values during the second month, and gradually rise in late lactation^42^.

In line with previous literature^43,44^, the increase in BILt and the reduction in albumin are associated with livers of larger size and with higher TAG contents. Low albumin values with high BILt values in early lactation are associated with the highest frequencies of metabolic and infectious diseases^45,46^ as well as the lowest production and fertility performances.

PON, an enzyme exclusively synthetized by the liver, hydrolyzes specific oxidized lipids leading to a reduction in oxidative stress^47^. A significant reduction in PON in plasma has been observed in dairy cows with severe inflammatory conditions after calving^48^. In our study, we found an interesting pattern in multiparous cows, where increasing liver dimensions (increased PVD and LD) were associated with decreasing PON concentrations.

In multiparous cows, the level of plasma oxidative stress metabolites ROMt and AOPP were associated with liver larger dimensions (PVA, PVD and LD), whereas low concentrations of FRAP were associated with higher PVD. An increase in AOPP and ROMt and a reduction in plasma FRAP are typical signs of non-alcoholic fatty liver disease in humans^49,50^.

However, in the absence of clinical disease (as in the present study), our results seem to confirm that a certain degree of inflammation and oxidative stress after calving is an adaptive rather than a pathological process^51^ and can also explain the contrasting patterns observed in primiparous and multiparous cows. The association between pTAG and FRAP was negative in multiparous cows, and positive in primiparous cows. The progressive accumulation of TAG into the liver could reduce its capacity to produce antioxidant species (hence the decline in FRAP in plasma), which might be associated with a greater susceptibility of multiparous cows to develop metabolic diseases.

For both parity classes, higher thiol groups concentration was associated with lower PVA values. Indeed, thiol groups are essential antioxidant molecules protecting the organism against the damaging effects of reactive oxygen species, and are well known to prevent vein hypertension in diverse pathologies in humans, especially portal hypertension^52,53^. The association between high levels of plasma antioxidants and small portal vein dimensions in our population provides further support for the crucial role of antioxidant systems in liver metabolic imbalance, and offers a potential means of monitoring liver oxidative stress processes to prevent disease in dairy cows.

Liver function can be affected by inflammation during the peripartum period^48,54^. Among the HC inflammation indicators, CP is a protein synthesized in the liver and the main carrier for copper (about 90%) in the blood. We found an association between increasing liver dimensions (PVD and LD) and increasing CP values, mainly in multiparous cows. In dairy cows, it has been found that during the peripartal period, an increase in positive acute-phase proteins (including CP) and a decrease in negative acute-phase proteins (*e.g.* albumin, lipoproteins) is often a physiological response to inflammation^45,48,55^. This can also explain the association between the increase in globulin levels (protein fraction, which also includes acute-phase proteins such as CP) and the increase in liver pTAG content. Moreover, in periparturient high-yielding dairy cows, higher CP correlates with higher interleukin-6 (IL-6)^45^, which seems to play a central role in the impairment of normal liver functions in transition cows^56^. The positive association between globulin and liver pTAG content might be explained by the contribution of lipomobilization and oxidative stress to the physiological inflammatory conditions occurring during early lactation.

Blood minerals are involved in many metabolic pathways. Changes in the plasma concentrations of minerals have been previously associated with fatty liver in dairy cattle; reduced concentrations of magnesium have been reported, as in the case of the multiparous cows in the present study^44,57^.

The level of zinc in plasma is mainly affected by inflammatory response: this mineral is sequestered into the liver tissue in the case of inflammation and its concentration in plasma is reduced^24^, even when adequately supplied in the diet^26^. observed accumulation of zinc in the liver of culled cows when plasma levels of zinc were below 11 μmol/l. The association between low plasma zinc levels (< 9 μmol/l) and high pTAG content in the liver therefore confirms this blood index as a good predictor of liver lipidosis, particularly in multiparous cows. More research is, however, needed to elucidate the association between mineral metabolism and liver health status.

In this study, we found that multiparous cows often exhibit different responses to primiparous cows due to their high productivity and stressful conditions which make them more susceptible to metabolic disorders, as previously reported^29^. The results obtained strengthen the evidence for a relationship between excessive lipomobilization, oxidative stress and dysfunctional inflammatory conditions in early lactation, which represent the nexus between metabolic and infectious diseases in dairy cattle^58^.

The findings of this study have to be considered in light of some limitations. First, our dataset did not comprise a consistent group of animals with evidence of metabolic disease, so our pattern of associations cannot be confirmed on animals with clinical disease. Second, liver biopsies were not available as a direct evaluation of TAG content; therefore, the combination of US and HC parameters cannot be proposed as a predictive tool for diagnosing subclinical condition of hepatic lipidosis as long as further validation with gold standard techniques will not be performed. Third, with our statistical approach we tested only associations between US and HC traits, and we did not explore the existence of potential cause-effect relationships which could be investigated in future studies.

In conclusion, we found several associations between US liver measurements, including texture analysis, and HC indicators of energy metabolism, liver oxidative stress and innate immune response in clinically healthy dairy cows. Moreover, we evidenced that variations in PVD and blood metabolites of liver function (*e.g.* AST/GOT, BILt, PON) seemed to be promising indicators of liver metabolic fluctuations.

Further validation on a larger sample size (including also pathological cases) are however needed to prompt the use of these indicators for the early detection of metabolic alterations in dairy cattle.

## Methods

### Animals and sampling

This study is part of a broader project aimed at devising new strategies for improving animal welfare in dairy cattle breeding, involving 1,038 Holstein Friesian dairy cows reared in two herds (herd A and herd B) located in Piacenza Province (northwestern Italy). The project was approved by the ethical committee of the OPBA—Organismo Preposto al Benessere degli Animali of the Università Cattolica del Sacro Cuore and by the Italian Ministry of Health (protocol number 510/2019-PR of 19/07/2019) and all methods were performed in accordance with the relevant guidelines and regulations. The animals in both herds were kept in a free stall housing system and fed on total mixed rations. The ingredients and chemical compositions of the diets are reported in Supplementary Table S2. Drinking water was available from automatic water bowls, and milking was carried out twice a day. Cows had an average daily milk yield of 37.49 kg/d (± 8.32) and an average body condition score of 3.10 (± 0.22). Body condition score was obtained according to^59^ classification on a scale ranging from 1 (emaciated) to 5 (extremely fat).

In the present study, we focused on cows in early lactation. Liver US measurements were taken from 342 clinically healthy primiparous and multiparous Holstein Friesian dairy cows (56 in herd A, 286 in herd B). Animals were defined “clinically healthy” by veterinary professionals on the absence of overt clinical and US signs of disease.

Blood samples (5 mL) from the jugular vein of 297 animals (35 in herd A, 262 in herd B) were collected in vacuum tubes containing 150 lithium heparin USP units (Vacumed; FL Medical, Torreglia, Padua, Italy). Blood sampling and liver US were carried out on the same day for each cow after the morning milking and before feeding from September 2019 to February 2020 (9 different herd/dates). For each herd/date, between 6 and 61 animals were sampled for US measurements, and between 8 and 58 for blood samples.

### Ultrasonographic evaluation

Transcutaneous US examination was carried out by a single operator (veterinarian highly experienced in liver US imaging) and using the same equipment and procedure as described in details^18^ which applied US to estimate the degree of fatty infiltration of the liver in 48 Holstein Friesian cows and validated this methodology with transcutaneous biopsies and complete histological examination. The regression equation used to predict the pTAG (mg/g) content of the liver was also taken from^18^. Briefly, Liver US examinations were performed on the right side of the animal kept in a standing position. The hepatic parenchyma was evaluated using a Mylab OneVET portable US scanner (Esaote SpA, Genoa, Italy) connected to a linear probe (Animal Science Probe, SV3L11; Esaote SpA, Genoa, Italy). The acoustic space for the penetration of the sound waves was located at the 10th intercostal space. The US frequency (2.8 MHz) and depth (21 cm) settings were kept constant for visualization of the hepatic parenchyma of all the animals enrolled in the study. The skin area selected for the US examination was degreased with 90% alcohol, cleaned with water, and smeared with US gel to improve the images. The probe was then moved dorsal-ventrally to an amplitude of about 15 cm in the intercostal space. The liver was also examined for the presence of focal lesions, including abscesses, neoplastic masses or abnormal lipid infiltrations. Multiple US images were archived in medicine format without compression (DICOM) for further analysis. The final US image for each animal was selected by a single operator based on its diagnostic capacity.

Liver depth (LD, mm), the portal vein area (PVA, mm2), and the portal vein depth (PVD, mm) were measured using the MyLab Desk software (Esaote SpA, Genoa, Italy), as reported by^20^. The hepatic parenchyma was analyzed using the MaZda v.4.6 texture analysis software (Technical University of Lodz, Institute of Electronics, Poland).

### Hematochemical parameters

After collection, the blood samples were kept in ice until centrifugation (Hettich Universal 16R Centrifuge, 3500 G, 16 min, 6 °C), which was performed within 2 h of collection. A small fraction of blood was used to determine hematocrit (packed cell volume) (ALC Centrifugette 4203, 15,300 G, 12 min). The plasma obtained from the centrifugation was stored at -20 °C until analysis. A clinical auto-analyzer (ILAB-650, Instrumentation Laboratory, Bedford, MA) was used to determine the concentrations of glucose, NEFA, BHBA, urea, creatinine, calcium, phosphorus, magnesium, sodium, potassium, chlorine, zinc, AST-GOT, GGT, alkaline phosphatase, total protein, haptoglobin, CP, albumin, BILt, cholesterol and globulin according to^60^; ROMt, FRAP and PON according to^61^; thiol groups according to^62^; myeloperoxidase according to^63^; and AOPP according to^64^*.*

### Statistical analyses

#### Exploratory data analysis

Initially, an exploratory data analysis was performed to check assumptions required for model fitting, hypothesis testing and handling extreme values. Specifically, the Shapiro–Wilk test was used to evaluate deviations from normal distribution for the pTAG, LD, PVA and PVD traits and for HC parameters. Skewness and kurtosis were also computed in order to characterize the distribution shape of response variables. Pearson correlations among HC traits were computed and visualized using the R *Hmisc* and *corrplot* packages in order to assess multicollinearity among traits that will be treated as predictors in subsequent analyses (Supplementary Figure S2). All statistical analyses were performed using the R software v. 3.6.3 (www.r-project.org).

#### Statistical inference

All the linear mixed models were implemented in the R *lme4* package. Before fitting the final model, which has been used to assess the association between pTAG, LD, PVA and PVD traits and HC traits, a preliminary analysis was conducted to test potential source of variation that might affect the US measurements. Indeed, a first base model was run to assess the sources of variation that might affect the US traits using the entire dataset:

$${\text{y}}_{ijkl} =\upmu + {\text{ DIM}}_{i} + {\text{Parity}}_{j} + \left( {{\text{DIM }} \times {\text{ Parity}}} \right)_{ij} + {\text{Herd}}/{\text{Date}}_{k} + {\text{e}}_{ijkl} , \quad \left[{ {{{\text{M-US}}}}} \right]$$

where y_*ijkl*_ is the observed trait (pTAG, LD, PVA and PVD); μ is the overall mean; DIM_*i*_ is the fixed effect of the *i*th class of days in milk (i = 5 classes; class 1 ≤ 30; 30 < class 2 ≤ 60; 60 < class 3 ≤ 90; 90 < class 4 ≤ 120; class 5 > 120); parity_*j*_ is the fixed effect of the *j*th parity (j = primiparous; multiparous); Herd/Date_*k*_ is the random effect of the *k*th herd/date (k = 1 to 10); and e_*ijkl*_ is the random residual. Herd/date and residuals were assumed to be normally distributed with a mean of zero and variances of ${\sigma }_{h}^{2}$ and ${\sigma }_{e}^{2}$, respectively. Restricted maximum likelihood was used as the method of estimation of variance components. The proportion of variance explained by herd/test date was calculated by dividing the corresponding variance component by the total variance.

A second model was fitted to investigate the association between the US traits and HC variables taking into consideration individual source of variations defined in the base model (M-US) and including animals for which blood samples were available (n = 297). To study this association, we adopted a conservative approach in which we did not assume any linear relationship between the response and independent variables. In order to correctly interpret the variations within the HC traits, the latter were discretized in classes according to physiological thresholds, as reported in Supplementary Table S3. Moreover, since DIM and parity affects the variability in HC traits^24,27,37^, the HC × DIM and HC × Parity interactions were also included. The DIM × Parity interaction, however, was not included since it was never significant.

Therefore, the resulting two models with increasing complexity were:

$$\begin{aligned} & {\text{y}}_{ijklm} =\upmu + {\text{DIM}}_{i} + {\text{Parity}}_{j} + {\text{HC}}_{k} + {\text{Herd}}/{\text{Date}}_{l} + {\text{e}}_{ijklm}\quad \left[ {\text{M-HC}} \right] \\ & {\text{y}}_{ijklm} = \,\upmu \, + {\text{ DIM}}_{i} + {\text{ Parity}}_{j} + {\text{ HC}}_{k} + \, \left( {{\text{HC }} \times {\text{ DIM}}} \right)_{ik} + \, \left( {{\text{HC }} \times {\text{ Parity}}} \right)_{jk} + {\text{ Herd}}/{\text{Date}}_{l} + {\text{ e}}_{ijklm} , \quad \left[ {\text{M-HC- int}} \right] \\ \end{aligned}$$

where y_*ijklm*_ is the observed trait (pTAG, LD, PVA and PVD); HC_*k*_ is the fixed effect of the *k*th class of HC described in Supplementary Table S3; parity_*j*_ is the fixed effect of the *j*th parity (j = 1; ≥ 2) and Herd/Date_*l*_ is the random effect of the *l*th herd/date (l = 1 to 9). All the other terms were as previously defined. In all the models tested, a given effect (or interaction) was declared significant at *P* < 0.05 and a tendency was considered if 0.05 ≤ *P* < 0.1. Only significant results or tendencies from the M-HC and M-HC-int models are displayed in Figs. 2, 3, 4, 5 and 6.

## Supplementary Information

Supplementary Information.

## Data Availability

The datasets generated and/or analyzed during the current study are available from the corresponding author on reasonable request.
