# Supplementary material for: Associations between ultrasound measurements and hematochemical parameters for the assessment of liver metabolic status in Holstein–Friesian cows
Source: Sci Rep. 2021 Aug 11;11:16314. doi: 10.1038/s41598-021-95538-x (PMC8357813; doi:10.1038/s41598-021-95538-x)
Supplement: Supplementary file 1 — Supplementary Information. [file 41598_2021_95538_MOESM1_ESM.docx]

**Associations between ultrasound measurements and hematochemical parameters for the assessment of liver metabolic status in Holstein-Friesian cows**

Diana Giannuzzi, Rossella Tessari, Sara Pegolo, Enrico Fiore, Matteo Gianesella, Erminio Trevisi, Paolo Ajmone Marsan, Michele Premi, Fiorenzo Piccioli-Cappelli, Franco Tagliapietra, Luigi Gallo, Stefano Schiavon, Giovanni Bittante and Alessio Cecchinato

**Supplementary Table S1.** Descriptive statistics of ultrasonographic and hematochemical indicators.

| Parameters | N | Mean | SD | P1^1^ | P99^1^ |
| --- | --- | --- | --- | --- | --- |
| *Hepatic ultrasonographic measures* |  |  |  |  |  |
| Predicted liver triacylglycerol (pTAG), mg/g | 338 | 69.52 | 10.85 | 49.92 | 99.65 |
| Portal vein area (PVA), mm^2^ | 330 | 1115.65 | 283.58 | 567.48 | 1864.4 |
| Portal vein depth (PVD), mm | 338 | 130.77 | 13.27 | 98.64 | 162.79 |
| Liver depth (LD), mm | 323 | 149.12 | 13.40 | 115.52 | 178.41 |
| *Hematochemical parameters* |  |  |  |  |  |
| Hematocrit, l/l | 296 | 0.30 | 0.02 | 0.24 | 0.37 |
| *Liver function/hepatic damage* |  |  |  |  |  |
| AST/GOT, U/l | 297 | 96.44 | 23.73 | 68.14 | 173.72 |
| GGT, U/l | 297 | 25.51 | 10.25 | 12.40 | 79.45 |
| Total bilirubin (BILt), μmol/l | 297 | 2.61 | 1.67 | 0.66 | 10.57 |
| Albumin, g/l | 297 | 36.99 | 2.32 | 30.28 | 41.76 |
| Alkaline phosphatase (ALP), U/l | 297 | 63.55 | 24.08 | 20.86 | 134.41 |
| Paraoxonase, U/ml | 297 | 105.10 | 21.00 | 54.45 | 158.78 |
| *Energy and protein related metabolites* |  |  |  |  |  |
| Glucose, mmol/l | 297 | 4.33 | 0.39 | 3.21 | 5.15 |
| Cholesterol, mmol/l | 297 | 4.63 | 1.25 | 1.82 | 7.84 |
| Nonesterified fatty acid (NEFA), mmol/l | 297 | 0.17 | 0.20 | 0.04 | 0.96 |
| β-hydroxybutyric acid (BHBA), mmol/l | 297 | 0.56 | 0.29 | 0.27 | 1.67 |
| Urea, mmol/l | 297 | 6.28 | 1.07 | 3.81 | 8.84 |
| Creatinine, μmol/l | 297 | 81.55 | 6.19 | 70.11 | 98.00 |
| *Inflammation/innate immunity* |  |  |  |  |  |
| Haptoglobin, g/l | 297 | 0.39 | 0.33 | 0.10 | 1.47 |
| Ceruloplasmin (CP), μmol/l | 297 | 1.84 | 0.61 | 0.78 | 3.65 |
| Total protein, g/l | 297 | 80.53 | 4.92 | 67.65 | 94.87 |
| Globulin, g/l | 297 | 43.55 | 5.28 | 34.14 | 57.70 |
| Mieloperoxidase, U/l | 297 | 453.4 | 69.31 | 285.13 | 656.15 |
| *Oxidative stress* |  |  |  |  |  |
| Total reactive oxygen metabolites (ROMt), mgH_2_O_2_/100ml | 297 | 12.60 | 3.17 | 5.86 | 20.33 |
| Advanced oxidation protein products (AOPP), μmol/l | 297 | 44.93 | 9.42 | 24.44 | 70.58 |
| Ferric reducing ability of plasma (FRAP), μmol/l | 297 | 195.14 | 69.75 | 124.23 | 354.20 |
| Thiol groups, μmol/l | 297 | 377.6 | 51.61 | 266.44 | 514.6 |
| *Minerals* |  |  |  |  |  |
| Calcium, mmol/l | 297 | 2.51 | 0.11 | 2.21 | 2.77 |
| Phosphorus, mmol/l | 297 | 1.93 | 0.34 | 1.07 | 2.76 |
| Magnesium, mmol/l | 297 | 0.99 | 0.09 | 0.71 | 1.20 |
| Sodium, mmol/l | 297 | 142.1 | 2.98 | 135.29 | 148.21 |
| Potassium, mmol/l | 297 | 4.08 | 0.41 | 3.07 | 5.04 |
| Chlorine, mmol/l | 297 | 101.33 | 3.25 | 92.60 | 108.37 |
| Zinc, μmol/l | 297 | 11.63 | 2.98 | 6.38 | 21.54 |

^1^ P1 = 1^st^ percentile; P99 = 99^th^ percentile.

**Supplementary Table S2**. Diet composition (% of DM) during lactation and dry period in the two herds.

|  |  | Herd A | |  | Herd B | | |
| --- | --- | --- | --- | --- | --- | --- | --- |
|  |  | Lactation | Dry |  | Lactation primiparous | Lactation multiparous | Dry |
| Corn silage |  | 12.42 | - |  | 30.76 | 32.94 | 31.49 |
| Sorghum silage |  | 24.6 | 26.61 |  | - | - | - |
| Wheat silage |  | - | - |  | 9.49 | 9.49 | - |
| Alfalfa hay |  | 20.98 | - |  | 11.47 | 11.48 | - |
| Ryegrass hay |  | 2.12 | 47.15 |  | - | - | - |
| Straw |  | - | 14.14 |  | - | - | 43.49 |
| Corn grain ground |  | 12.62 | - |  | 23.95 | 21.16 | 2.00 |
| Barley grain ground |  | 8.41 | - |  | - | - | - |
| Soybean meal |  | 12.1 | 5.53 |  | 9.25 | 10.65 | 10.01 |
| Sunflower meal |  | 4.34 | 5.78 |  | 4.69 | 4.43 | 2.54 |
| Soybean |  | - | - |  | 3.52 | 3.32 | - |
| Wheat midds |  | - | - |  | 4.58 | 4.32 | 9.48 |
| Hydrogenated fat |  | 0.78 | - |  | 0.55 | 0.52 | - |
| Minerals and vitamin supplements^1^ |  | 1.63 | 0.79 |  | 1.76 | 1.69 | 1.00 |
|  |  |  |  |  |  |  |  |
| *Nutrient composition* |  |  |  |  |  |  |  |
| UFL (U/kg DM) |  | 0.97 | 0.78 |  | 0.98 | 0.98 | 0.72 |
| ENl (Mcal/kg DM) |  | 1.55 | 1.31 |  | 1.59 | 1.53 | 1.15 |
| Crude protein (% DM) |  | 16.50 | 12.50 |  | 15.97 | 16.34 | 11.51 |
| Metabolizable protein (% DM) |  | 10.50 | 8.27 |  | 10.79 | 10.92 | 8.24 |
| NSC (% DM) |  | 36.80 | 19.00 |  | 44.07 | 43.06 | 21.41 |
| NDF (% DM) |  | 35.80 | 56.00 |  | 32.18 | 32.91 | 58.68 |

^1^ Herd A: during the dry period dairy cows received 70 g/d of a supplement contained 42.9% Ca_2_PO_4_; 28.6% urea; 14.3% MgO; 7.1% NaCl; 7.1% mineral vitamin supplement composited to provide 100000 UI of vitamin A, 10000 IU of vitamin D, 500 mg of vitamin E, 100 mg of Mn, 300 mg of Zn, 50 mg of Cu, 5 mg of I, 1 mg of Co, 3 mg of Se. During the lactation dairy cows received 300 g/d of a supplement contained, 27.5% NaHCO_3_, 20% CaCO_3_, 20% CaHPO_4_; 7% MgO; 2% NaCl; 10% mineral vitamin supplement composited to provide, 150000 UI of vitamin A, 15000 IU of vitamin D, 1000 mg of vitamin E, 100 mg of vitamin K, 100 mg of vitamin H1 50 mg of vitamin B1, 0.5 mg of vitamin B12, 500 mg of vitamin PP, 4000 mg of choline, 700 mg of Mn, 1200 mg of Zn, 200 mg of Cu, 20 mg of I, 2 mg of Co, 4 mg Se.

Herd B: during the lactation dairy cows received a supplement composited to provide 132000 UI of vitamin A, 33000 IU of vitamin D, 650 mg of vitamin E, 770 mg of Mn, 1100 mg of Zn, 180 mg of Cu, 22 mg of I, 2.5 mg of Co, 3.6 mg of Se. During the dry period dairy cows received a supplement composited to provide 210000 UI of vitamin A, 54000 IU of vitamin D, 1000 mg of vitamin E, 1200 mg of Mn, 600 mg of Zn, 300 mg of Cu, 35 mg of I, 4.2 mg of Co, 5.5 mg of Se.

**Supplementary Table S3.** Hematochemical parameters discretized in classes.

| Hematochemical indicators | Classes | | | |
| --- | --- | --- | --- | --- |
|  | 1 | 2 | 3 | 4 |
| Hematocrit^1^, l/l | < 0.27 | 0.27 – 0.31 | > 0.31 | -- |
| *Liver function/hepatic damage* |  |  |  |  |
| AST/GOT^1^, U/l | < 87.6 | ≥ 87.6 | -- | -- |
| GGT^1^, U/l | < 25.3 | - 1. –34.5 | > 34.5 | -- |
| Total bilirubin^1^ (BILt), μmol/l | < 4.3 | ≥ 4.3 | -- | -- |
| Albumin^1^, g/l | < 33.3 | 33.3 – 35.0 | > 35.0 | -- |
| Alkaline phosphatase^1^ (ALP), U/l | < 34.5 | 34.5 – 55.8 | > 55.8 | -- |
| Paraoxonase^2^, U/ml | < 61.7 | 61.7 – 142 | > 142 | -- |
| *Energy and protein related metabolites* |  |  |  |  |
| Glucose^1^, mmol/l | < 3.67 | 3.6 – 4.08 | > 4.08 | -- |
| Cholesterol^3^, mmol/l | < 3.0 | 3.0 – 3.84 | 3.85 – 6.5 | > 6.5 |
| Nonesterified fatty acid^1^ (NEFA), mmol/l | < 0.12 | 0.12 – 0.38 | > 0.38 | -- |
| β-hydroxybutyric acid^1^ (BHBA), mmol/l | < 0.45 | 0.45 – 1.2 | > 1.2 | -- |
| Urea^1^, mmol/l | < 4.41 | 4.41 – 5.97 | > 5.97 | -- |
| Creatinine^1^, μmol/l | < 82.8 | 82.8 –98.5 | > 98.5 | -- |
| *Inflammation/innate immunity* |  |  |  |  |
| Haptoglobin^2^, g/l | < 0.30 | ≥ 0.30 | -- | -- |
| Ceruloplasmin^1^ (CP), μmol/l | < 2.19 | 2.19 –3.15 | > 3.15 | -- |
| Total protein^1^, g/l | < 77.0 | 77.0 – 84.9 | > 84.9 | -- |
| Globulin^1^, g/l | < 41.5 | 41.5 – 51.2 | > 51.2 | -- |
| Mieloperoxidase^2^, U/l | < 336 | 336 – 566 | > 566 | -- |
| *Oxidative stress* |  |  |  |  |
| Total reactive oxygen metabolites^2^ (ROMt), mgH_2_O_2_/100ml | < 19.6 | ≥ 19.6 | -- | -- |
| Advanced oxidation protein products^3^ (AOPP), μmol/l | 23.6 – 39.5 | 39.6 – 44.7 | 44.8– 50.2 | 50.3– 91.4 |
| Ferric reducing ability of plasma^2^ (FRAP), μmol/l | < 105 | 105 – 193 | > 193 | -- |
| Thiol groups^2^, μmol/l | < 221 | 221 – 480 | > 480 | -- |
| *Minerals* |  |  |  |  |
| Calcium^1^, mmol/l | < 2.38 | 2.38 – 2.59 | > 2.59 | -- |
| Phosphorus^1^, mmol/l | < 1.57 | 1.57 – 2.17 | > 2.17 | -- |
| Magnesium^1^, mmol/l | < 0.91 | 0.91 –1.03 | > 1.03 | -- |
| Sodium^1^, mmol/l | < 141.1 | 141.1–145.5 | > 145.5 | -- |
| Potassium^1^, mmol/l | < 3.67 | 3.67 – 4.64 | > 4.64 | -- |
| Chlorine^1^, mmol/l | < 100.2 | 100.2–106.6 | > 106.6 | -- |
| Zinc^1^, μmol/l | < 9 | 9 – 13 | > 13 | -- |

^1^Classes were created based on Bertoni and Trevisi (2013)

^2^Classes were created based on Premi (2020)

^3^Classes were created based on quartiles

**
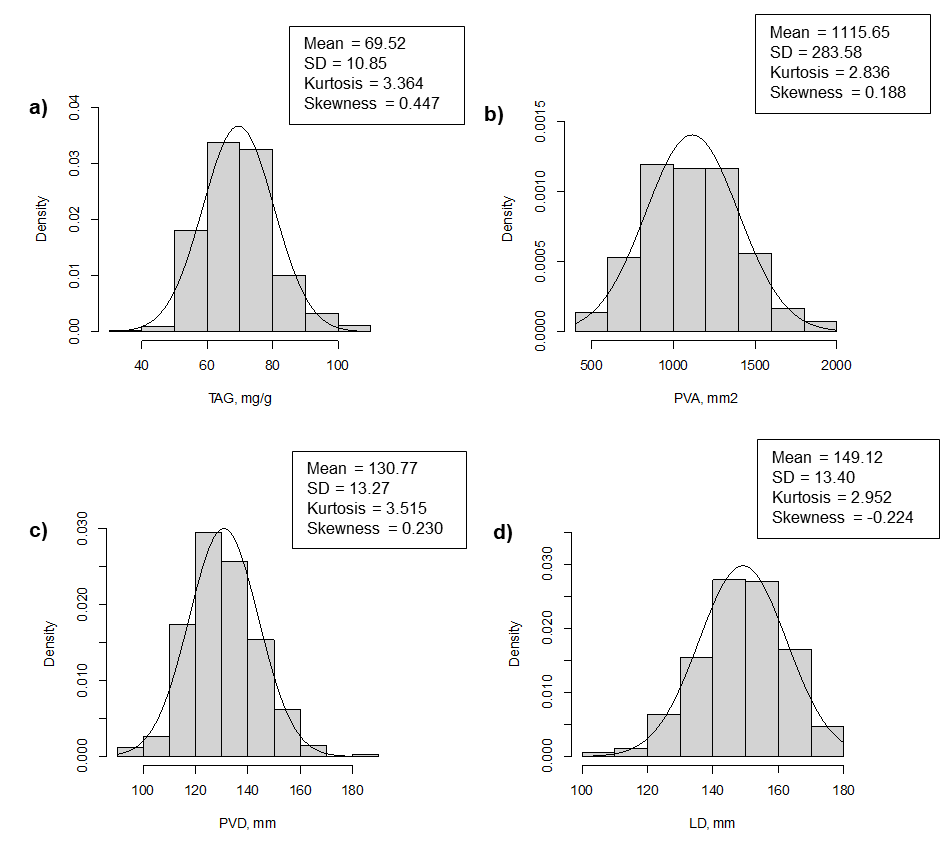
**

**Supplementary Figure S1. Distribution, mean and standard deviation (SD) values of a) triglycerides (TAG), b) portal vein area (PVA), c) portal vein depth (PVD) and d) liver depth (LD).** Kurtosis was calculated using Pearson’s method. Graphics have been created using the *Hmisc* R package within the R software v. 3.6.3 ([www.r-project.org](http://www.r-project.org)).
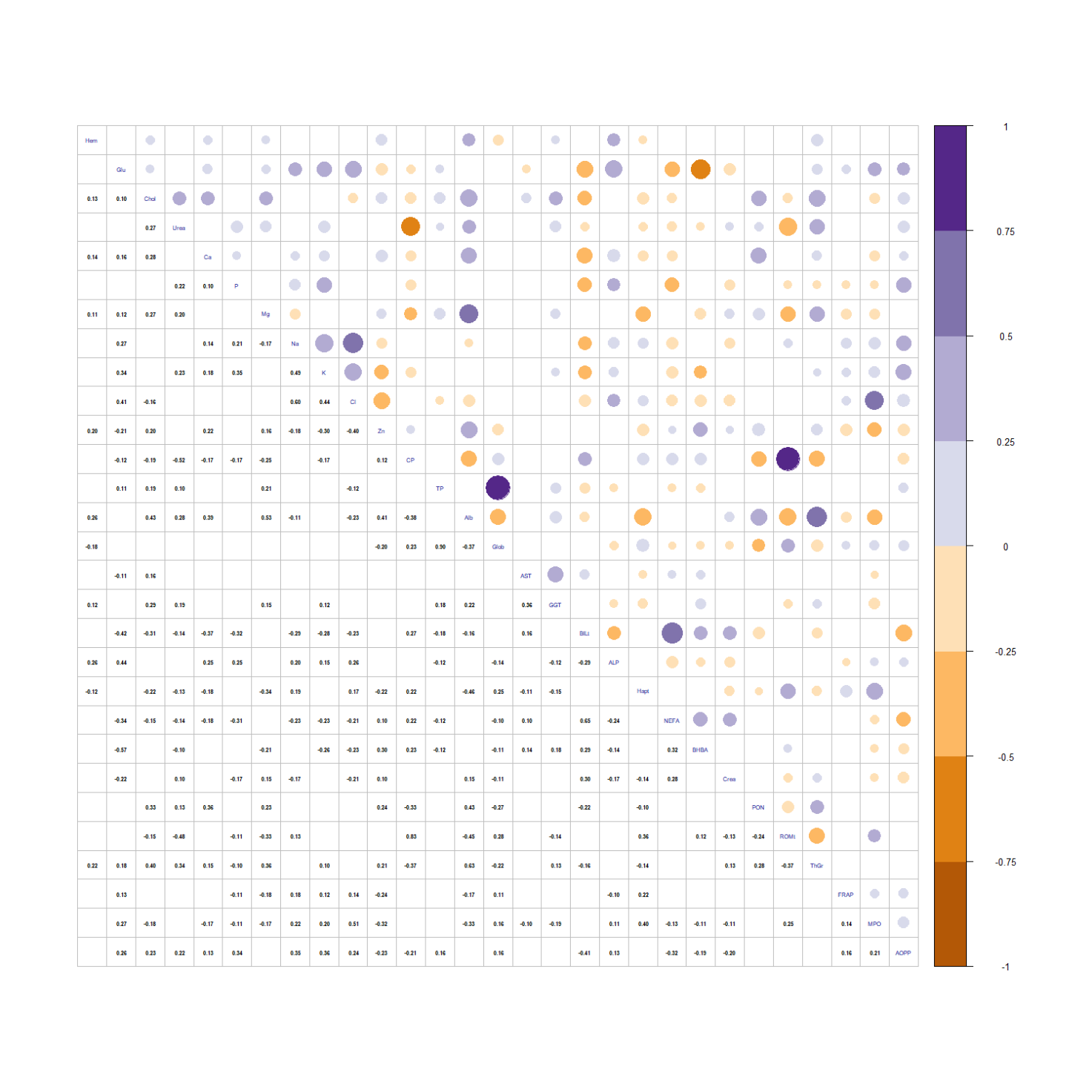
**Supplementary Figure S2.** Pearson’s correlations heatmap of hematochemical parameters. Red circles correspond to negative correlations and blue circles to positive correlations. The dimension of the circles is proportional to the strength of the correlation. Only correlations with P < 0.1 are displayed. Hem: hematocrit; Glu: glucose; Chol: cholesterol; CP: ceruloplasmin; TP: total protein; Glob: glubulins; AST: aspartate amino transferase- glutamate oxaloacetate transaminase; GGT: γ-glutamyl transferase; BILt: total bilirubin; ALP: alkaline phosphatase; Hapt: haptoglobin; NEFA: non-esterified fatty acids; BHBA: β-hydroxybutyric acid; Crea: creatinine; PON: paraoxonase; ROMt: reactive oxygen metabolites; ThGr: thiol groups; FRAP: ferric-reducing antioxidant power; MPO: myeloperoxidase; AOPP: advanced oxidation protein products. The correlation plot has been created using the *corrplot* R package within the R software v. 3.6.3 ([www.r-project.org](http://www.r-project.org)).
